# Development of a TaqMan One-Step Quantitative PCR Assay for the Simultaneous Detection of Novel Goose Parvovirus and Novel Duck Reovirus

**DOI:** 10.3390/microorganisms13071582

**Published:** 2025-07-04

**Authors:** Yimin Wang, Yong Wang, Zhuangli Bi, Jinbin Wang, Gang Wang, Xin Ru, Chunchun Meng, Jie Zhu, Guangqing Liu, Chuanfeng Li

**Affiliations:** 1 Shanghai Veterinary Research Institute, Chinese Academy of Agricultural Sciences (CAAS), Shanghai 200241, China; incir0204@163.com (Y.W.); bzl601926835@163.com (Z.B.); mengcc@shvri.ac.cn (C.M.); zj121@shvri.ac.cn (J.Z.); 2 College of Animal Science and Technology, Anhui Agricultural University, Hefei 230036, China; wangyong119@ahau.edu.cn (Y.W.); aisi975@outlook.com (J.W.); 17368836936@139.com (G.W.); ruxin9908@outlook.com (X.R.)

**Keywords:** novel goose parvovirus, novel duck reovirus, simultaneous detection, one-step qPCR

## Abstract

The novel goose parvovirus (NGPV) and the novel duck reovirus (NDRV) are pathogens that can substantially affect the growth and development of ducklings, causing considerable economic losses to duck farms. Therefore, a timely, rapid, accurate, and high-throughput diagnosis and identification of viral infections are critical for preventing the spread of epidemics. In this study, a TaqMan probe-based duplex one-step RT-qPCR was established for the simultaneous detection and qualitative and quantitative identification of the two viruses. It demonstrated greater sensitivity than conventional PCR, detecting as low as 2.42 copies/μL of NGPV genome and 70.1 copies/μL of NDRV genome. Additionally, it exhibited remarkable specificity, responding exclusively to the nucleic acids of target pathogens. It also demonstrated excellent reproducibility and availability, particularly in clinical settings, with a coinfection detection rate of 13.3%, contributing to the development of NGPV- and NDRV-testing technologies.

## 1. Introduction

In 2016, the novel goose parvovirus (NGPV) was first identified as the causative agent of short beak and dwarfism syndrome, which is characterized predominantly by tongue protrusion, short beak, and growth retardation [1,2]. This virus primarily induces a 20–30% reduction in the weight of infected ducks compared to their healthy counterparts [3]. Cases of NGPV infections have been documented in numerous countries with a prevalence of up to 60%, albeit with relatively low mortality rates [1,4,5,6]. In contrast, infection with the novel duck reovirus (NDRV) has been demonstrated to result in a significantly higher mortality rate, reaching up to 84% [7]. The two principal clinicopathological changes observed in ducks infected with the virus are hemorrhagic necrotizing hepatitis and splenic necrosis [8,9]. NGPV is a small single-stranded DNA virus (family Parvoviridae), while NDRV is a double-stranded RNA virus (family Reoviridae), belonging to different viral families. The non-structural protein 1 (*NS1*) of NGPV is a conserved replication initiator, exhibiting high amino acid homology across strains, making it ideal for broad viral screening. In contrast, the *σB* gene of NDRV encodes the major capsid protein, which contains genotype-specific epitopes enabling subtype discrimination. The dual-target design permits simultaneous screening for two virus types, which is achieved via *NS1* and *σB* markers. These two viral infections can have a significant impact on the growth and health of ducklings, resulting in considerable economic losses to the duck farming industry [2,10]. Therefore, timely and accurate diagnosis and identification are crucial in controlling the spread of these two viral infections and preventing the spread of epidemics.

The current testing methods for these viruses include virus isolation, serological assays, and molecular biology techniques [11,12,13]. The first two methods, which include enzyme-linked immunosorbent assay (ELISA), are time-consuming and complex [11,14]. The loop-mediated isothermal amplification (LAMP) assay is a rapid and convenient method; however, it is prone to false-positive results [13,15]. Other detection methods, such as recombinase polymerase amplification (RPA) and clustered regularly interspaced short palindromic repeats (CRISPR)/CRISPR-associated nuclease (CRISPR/Cas) systems, require specialized reagents and are associated with higher costs, making them less suitable for small laboratory settings [16,17]. The quantitative real-time polymerase chain reaction (qPCR) is regarded as the gold standard for pathogen detection. Several qPCR methods have been developed for detecting NGPV or NDRV infections [18,19,20]. It is essential to acknowledge that none of the above methods can detect both viruses simultaneously. Recently, a SYBR Green Ι-based duplex qPCR assay which can detect both NGPV and NDRV simultaneously has been developed in our laboratory [21]. It still employed the traditional two-step approach, which is complicated by the need to prepare templates from different types of nucleic acids. Furthermore, this method only achieved simultaneous qualitative detection for co-infection with two viruses. Therefore, in this study, a one-step duplex reverse transcription qPCR (RT-qPCR) method based on a TaqMan probe was established to achieve simultaneous detection of the two viruses qualitatively and quantitatively. This method has been verified by specificity, sensitivity, and repeatability assays and provides a rapid and accurate tool for distinguishing pathogens and for epidemiological investigations.

## 2. Materials and Methods

The NGPV (DS15 strain, KX384726.2) and NDRV (TH11 strain, JX826587.1) viruses used in this study were isolated from China. For specificity analysis, avian influenza virus [AIV, A/duck/Shanghai/29-1/2009 (H4N2) strain, KX162615.1], duck flavivirus (DFV, FX2010 strain, MH414568.1), duck hepatitis A virus (DHAV, ZJ strain, EF382778.1), Newcastle disease virus (NDV, Du/CH/SD/2009/134 strain, KJ600786.1), and duck enteritis virus (DEV, VAC strain, EU082088.2) were used. All the specimens were stored at the Companion Animal Biosecurity Risk Early Warning, Prevention and Control Technology Team, Shanghai Veterinary Research Institute, Chinese Academy of Agricultural Sciences (CAAS). For each specimen, 1 g of clinical tissue (the mixture of heart, liver, spleen, lungs, kidneys, and other organs) was added to 1 mL of phosphate-buffered saline (PBS) at pH 7.0, crushed thoroughly with a grinder (Thermo Fisher Scientific, Waltham, MA, USA) and centrifuged at 12,000 rpm for 5 min (Eppendorf, Hamburg, Germany). The supernatant was used for the extraction of nucleic acids. In the case of harvested swabs, each specimen containing 500 μL of PBS was subjected to nucleic acid extraction following a straightforward centrifugation process to remove any impurities. The total nucleic acids (viral DNA and RNA) were extracted using the TIANamp Virus DNA/RNA Kit (Tiangen, Beijing, China) according to the manufacturer’s instructions. The RNA was reverse-transcribed to cDNA using the PrimeScript™ II 1st Strand cDNA Synthesis Kit (Vazyme, Nanjing, China). All the nucleic acids were stored at −80 °C and used for subsequent experiments.

The NGPV DNA and NDRV cDNA were used as templates for constructing standard plasmids. The primers used for the PCR amplification of NGPV and NDRV nucleic acids were designed using the Primer Premier 5 software, based on the highly conserved regions of the *NS1* and the *σB* genes, respectively. The PCR product generated from the forward primer NGPV-F (5′-AGAGTGATTTGGCTGCCCCT-3′) and the reverse primer NGPV-R (5′-GCTTCTGTAGCTGTCTCGGTTCT-3′) was 167 base pairs (bp) in size, while the PCR product generated from the forward primer NDRV-F (5′-TGCCATCGCTCTGCATGTC-3′) and the reverse primer NDRV-R (5′-CCAACCCGCTCTAGCAATCTC-3′) was 211 bp in size. After purification, the two PCR products were cloned into the pEASY vector (TransGen, Beijing, China) and transformed into E.coli DH5α competent cells (Sangon, Shanghai, China), for plasmid extraction. Thereafter, sequencing of the plasmids was carried out by Huagene Biotech (Shanghai, China), and the correct recombinant plasmid of NGPV was directly used in subsequent experiments. The recombinant plasmid of NDRV was linearized with Spe I (Takara, Dalian, China), transcribed into RNA using the T7 High Yield RNA Transcription Kit (Vazyme, Nanjing, China), and then purified using the EasyPure^®^ RNA Purification Kit (TransGen, Beijing, China) according to the manufacturer’s instructions. The concentrations of both DNA and RNA standards were measured using a Nanodrop (Thermo Fisher Scientific, Waltham, MA, USA), yielding copy numbers of 2.42 × 10^10^ copies/μL and 7.01 × 10^9^ copies/μL, respectively.

A duplex one-step RT-qPCR assay was developed for the simultaneous detection of NGPV and NDRV. The specific primers described above, which were also employed in the qPCR assay, encompassed the genomic regions of NGPV-F and NGPV-R, as well as that of NDRV-F and NDRV-R. The TaqMan probe for NGPV (5′-TGTCGCGCGTCGTATCCTCGCCTCT-3′) was labeled with the fluorophore JOE at the 5′ end and the quencher BHQ1 at the 3′ end. The TaqMan probe for NDRV (5′-ATCAACTGTGGCACCGCTTATTTCC-3′) was labeled with the fluorophore FAM at the 5′ end and the quencher BHQ1 at the 3′ end. All the primers and probes were synthesized by Huagene Biotech (Shanghai, China). The duplex one-step RT-qPCR assay was performed using a StepOnePlus real-time PCR system (Applied Biosystems, Waltham, MA, USA) and TransScript^®^ II Multiplex Probe One-Step qRT-PCR SuperMix UDG (TransGen, Beijing, China).

## 3. Results and Discussion

To develop the assay, the annealing temperatures (56–64 °C) and the concentrations of the primer set (0.1–1.0 µM) and probes (0.1–0.3 µM) were optimized through gradient experiments. The optimal combination was selected based on three criteria: specific amplification (absence of primer dimers or spurious bands), the lowest Ct value, and the maximal fluorescence intensity. The optimal reaction system had a total volume of 20 μL, comprising 10 μL of 2 × PerfectStart^®^ Multiplex Probe One-Step Reaction Mix, 0.8 μL of TransScript^®^II Multiplex Probe One-Step Enzyme Mix UDG, 0.4 μL of Passive Reference Dye I, 0.4 μL each of forward and reverse primers for NGPV, 1.4 μL each of forward and reverse primers for NDRV, 0.2 μL of each probe for NGPV and NDRV, 2 μL of template, and sterilized ddH_2_O to a final volume of 20 μL. The optimal reaction conditions were as follows: 50 °C for 5 min, followed by 94 °C for 30 s, and then 40 cycles of 94 °C for 5 s and 58 °C for 30 s. At the end of the reaction, Ct values were automatically calculated by the qPCR instrument. Each reaction was conducted in triplicates, with ddH_2_O as the negative control.

**Figure 1:**
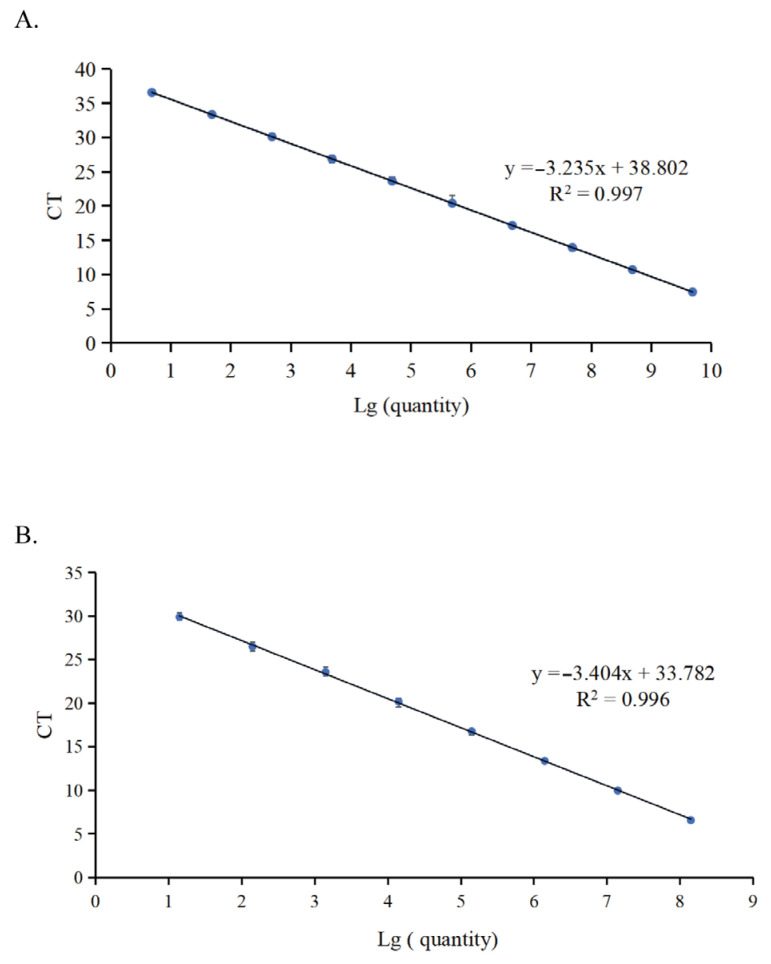
The standard curve of the duplex one-step qPCR assay. (**A**) The standard curve of NGPV generated by duplex one-step qPCR was established with serial 10-fold diluted DNA standards, including 2.42 × 10^9^ to 2.42 × 10^0^ copies/μL. (**B**) The standard curve of NDRV generated by duplex one-step qPCR was established with serial 10-fold diluted RNA standards, including 7.01 × 10^8^ to 7.01 × 10^1^ copies/μL.

To evaluate the amplification efficiency, the DNA/RNA standards were serially diluted 10-fold from 10^9^ to 10^0^ copies/μL and used to establish standard curves. The curves were plotted with the triplicate mean Ct value as the vertical coordinate and the concentrations of the standard plasmid as the horizontal coordinate. The linear equation of the regression curve (y) and square of the correlation coefficient (R^2^) were automatically generated by the fluorescence machine. The standard curve, R^2^, and amplification efficiency for NGPV were y = −3.235x + 38.802, 0.997, and 103%, respectively (Figure 1A). The standard curve, R^2^, and amplification efficiency for NDRV were y = −3.404x + 33.782, 0.996, and 96%, respectively (Figure 1B). The assay demonstrated a high degree of correlation and amplification efficiency, allowing for efficient detection of NGPV and NDRV.

**Figure 2:**
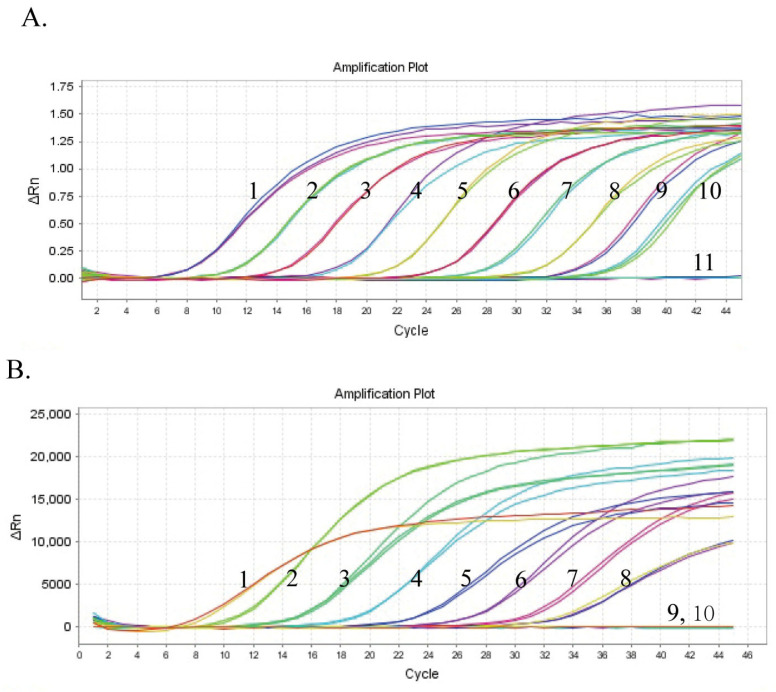
The sensitivity analysis of the duplex one-step qPCR assay. (**A**) Amplification curve of duplex one-step qPCR using the standard plasmids of NGPV. Numbers 1–10 represented NGPV DNA standards at different concentrations (serially ranging from 2.42 × 10^9^ to 2.42 × 10^0^ copies/μL) and 11 represented the negative control. (**B**) Amplification curve of duplex one-step qPCR using the NDRV RNA standards. Numbers 1–9 represented NDRV RNA standards at different concentrations (serially ranging from 7.01 × 10^8^ to 7.01 × 10^0^ copies/μL) and 10 represented the negative control.

To evaluate the sensitivity of the assay, the standards were diluted 10-fold serially from 10^9^ to 10^0^ copies/μL to determine the limit of detection (LOD). Each concentration of the specimen, including the negative control, was tested thrice. As illustrated in Figure 2A and 2B, the LODs of the duplex one-step RT-qPCR were 2.42 and 70.1 copies/μL for NGPV and NDRV, respectively.

**Figure 3:**
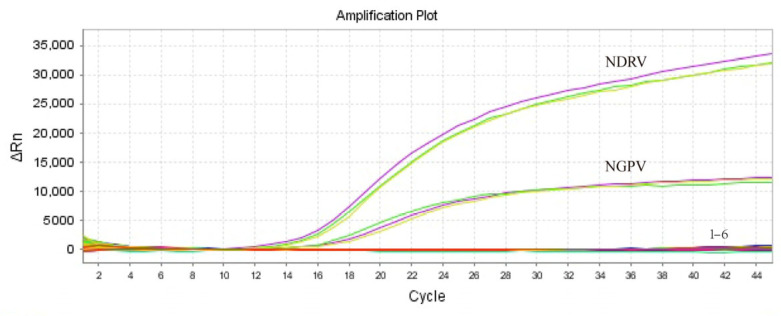
The specificity amplification curve of the NGPV and NDRV testing with the duplex one-step qPCR assay. Numbers 1–6 represent AIV, DHAV-3, DFV, NDV, DEV, and the negative control (ddH_2_O).

To assess the specificity of the assay, the positive nucleic acid templates of NGPV and NDRV (the concentrations at 10^6^ copies/μL for each pathogen) were used as positive controls, whereas the nucleic acids from the positive samples for five additional duck viral pathogens (AIV, DFV, DHAV, NDV, and DEV) were used as templates. Additionally, ddH_2_O was used as the negative control. The results showed that only the target nucleic acids were successfully amplified, demonstrating the high specificity of the TaqMan probe-based assay (Figure 3).

To assess the repeatability and reproducibility of the assay, the recombinant standard plasmids were diluted at three different concentrations (10^4^, 10^5^, and 10^6^ copies/μL) and selected as templates for the repeatability assay. The intra-assay reliability was evaluated by simultaneously assessing three replicates for each of the three plasmid dilutions. The inter-assay reliability was assessed by repeating three independent experiments every alternate week. As shown in Table 1 and Table 2, the intra- and inter-assay coefficients of variation were less than 1.1%, indicating that the assay exhibited good reproducibility.

To further validate the applicability of the duplex one-step RT-qPCR assay, a total of 75 clinical tissue or swab samples were analyzed, which were collected from cherry valley ducks suspected of NGPV or NDRV infection across multiple farms. Using the above-optimized reaction conditions, the samples were tested, and viral positivity was determined based on amplification curves in distinct fluorescence channels (JOE for NGPV and FAM for NDRV). The duplex one-step RT-qPCR assay identified 34 samples as positive (45.3%) and 41 as negative (54.7%) for NGPV, whereas 29 samples were positive (38.7%) and 46 as negative (61.3%) for NDRV. All the positive PCR products were verified by sequencing. Additionally, 75 clinical samples were assayed using conventional PCR, from which we found that the positive detection rates were 40% (30/75) for NGPV and 22.7% (17/75) for NDRV. In particular, the duplex one-step RT-qPCR assay identified co-infection with both the viruses in 10 cases (13.3%). However, no coinfected samples were detected using the conventional PCR assay. These data indicate that the duplex one-step RT-qPCR assay is more sensitive than the conventional PCR assay.

The novel qPCR assay developed in this study can enhance the detection capabilities for NGPV and NDRV. The method permitted the direct detection of viral total nucleic acids, thereby simplifying the experimental procedures involved. Furthermore, the entire time of the amplification reaction was under 1 h, in contrast to the 1.5 h required for the two-step qPCR. Significantly, the LOD was reduced by at least 10-fold due to the introduction of probes specifically targeted the viral genes compared with the SYBR Green Ι-based duplex qPCR assay [21]. In particular, it could facilitate the concurrent measurement of individual viral loads in instances where the co-infection of two viruses is detected. Because the TaqMan probe-based qPCR employs two distinct and non-interfering fluorescence channels to obtain signals, which are then used to generate two amplification curves. Whereas, SYBR Green Ι-based qPCR assay employs a shared channel, yielding a single Ct value for the duplex amplification curve, which does not accurately reflect the actual viral loads for either of the two viruses. Although this Taqman-based duplex detection method enables rapid qualitative and quantitative viral detection, several limiting factors merit consideration. First, the co-amplification efficiency may be compromised due to differing optimal denaturation temperatures for DNA (95 °C) and RNA (90 °C), potentially reducing the sensitivity of one target. Furthermore, it remains unclear whether differential nucleic acid inputs (DNA vs. RNA) might induce competitive amplification interference when analyzing complex clinical specimens.

Therefore, this study, a TaqMan probe-based qPCR was used to establish a detection assay for NGPV and NDRV infections, which can be carried out in most laboratories. It showed good specificity, based on two specific probes that exclusively amplified the target viruses. More importantly, the one-step qPCR reduced the total detection time and LODs while preserving good amplification efficiency. Moreover, its repeatability and practicality were valid. Its ability to detect and identify two viruses simultaneously makes it a rapid and effective tool in the field of virus detection.

## Figures and Tables

**Table 1:** Repeatability analysis results of duplex one-step qPCR for detecting NGPV.

Category	Concentration (Copies/μL)	Mean Ct	SD	CV (%)
Intra-assay	10^4^	24.130	0.074	0.32
	10^5^	20.919	0.052	0.26
	10^6^	17.116	0.024	0.14
Inter-assay	10^4^	24.863	0.043	0.17
	10^5^	20.981	0.055	0.26
	10^6^	17.319	0.108	0.62

SD: stand deviation; CV (%): coefficient of variation.

**Table 2:** Repeatability analysis results of duplex one-step qPCR for detecting NDRV.

Category	Concentration (Copies/μL)	Mean Ct	SD	CV (%)
Intra-assay	10^4^	20.510	0.057	0.27
	10^5^	16.847	0.144	0.85
	10^6^	13.060	0.072	0.55
Inter-assay	10^4^	20.110	0.160	0.79
	10^5^	16.391	0.073	0.44
	10^6^	12.840	0.136	1.05

SD: stand deviation; CV (%): coefficient of variation.

## Data Availability

The original contributions presented in this study are included in the article. Further inquiries can be directed to the corresponding authors.
